# Enhancing spiking neural networks with hybrid top-down attention

**DOI:** 10.3389/fnins.2022.949142

**Published:** 2022-08-22

**Authors:** Faqiang Liu, Rong Zhao

**Affiliations:** Department of Precision Instrument, Center for Brain-Inspired Computing Research, Beijing Advanced Innovation Center for Integrated Circuits, Tsinghua University, Beijing, China

**Keywords:** spiking, neuromorphic, hybrid, top-down, attention, robustness, efficiency

## Abstract

As the representatives of brain-inspired models at the neuronal level, spiking neural networks (SNNs) have shown great promise in processing spatiotemporal information with intrinsic temporal dynamics. SNNs are expected to further improve their robustness and computing efficiency by introducing top-down attention at the architectural level, which is crucial for the human brain to support advanced intelligence. However, this attempt encounters difficulties in optimizing the attention in SNNs largely due to the lack of annotations. Here, we develop a hybrid network model with a top-down attention mechanism (HTDA) by incorporating an artificial neural network (ANN) to generate attention maps based on the features extracted by a feedforward SNN. The attention map is then used to modulate the encoding layer of the SNN so that it focuses on the most informative sensory input. To facilitate direct learning of attention maps and avoid labor-intensive annotations, we propose a general principle and a corresponding weakly-supervised objective, which promotes the HTDA model to utilize an integral and small subset of the input to give accurate predictions. On this basis, the ANN and the SNN can be jointly optimized by surrogate gradient descent in an end-to-end manner. We comprehensively evaluated the HTDA model on object recognition tasks, which demonstrates strong robustness to adversarial noise, high computing efficiency, and good interpretability. On the widely-adopted CIFAR-10, CIFAR-100, and MNIST benchmarks, the HTDA model reduces firing rates by up to 50% and improves adversarial robustness by up to 10% with comparable or better accuracy compared with the state-of-the-art SNNs. The HTDA model is also verified on dynamic neuromorphic datasets and achieves consistent improvements. This study provides a new way to boost the performance of SNNs by employing a hybrid top-down attention mechanism.

## 1. Introduction

With complex structures and advanced cognitive functions, the human brain is a valuable reference for building artificial general intelligence. As the representatives of mimicking the brain at the neuronal level, spiking neural networks (SNNs) (Maass, 1997) have rich coding schemes and complex dynamics, which are suitable for processing spatiotemporal information (Tavanaei et al., 2019). With the support of neuromorphic hardware (Painkras et al., 2013; Benjamin et al., 2014; Schuman et al., 2017; Pei et al., 2019), event-driven updates and sparse spike emissions of SNNs lead to high energy efficiency and low inference latency. SNNs can be optimized by biologically inspired synaptic plasticity, such as the Hebbian learning rule (Gerstner and Kistler, 2002) and its variants (Masquelier and Thorpe, 2007). On the other hand, driven by the deep learning paradigm, SNNs can be directly learned based on surrogate gradient descent in an end-to-end manner (Wu et al., 2018, 2019; Neftci et al., 2019), or converted from pretrained deep artificial neural networks (ANNs) (Cao et al., 2015; Bu et al., 2021; Li et al., 2021). Recently, the development of hybrid networks by combining ANNs and SNNs has attracted increasing research interest (Pei et al., 2019; Lee et al., 2020; Wu et al., 2022). Hybrid networks leverage the unique characteristics of both parties, providing more diverse building blocks and flexible structures for supporting advanced intelligence.

In addition to the neuronal level, it is also important to draw inspiration from the brain at the architectural level. Top-down attention supported by ubiquitous feedback connections (Connor et al., 2004; Noudoost et al., 2010; Baluch and Itti, 2011) in the brain can select a subset of sensory input for processing according to internal goals or working memory (Corbetta and Shulman, 2002; Gazzaley and Nobre, 2012), facilitating the brain to concentrate on the most crucial information with limited cognitive processing resources (Desimone and Duncan, 1995). In the deep learning community, top-down attention has been investigated to improve the performance of ANNs on image classification tasks (Harris et al., 2019), and reinforcement learning is deployed to guide attention generation (Ba et al., 2015). Moreover, as revealed by findings in neuroscience, the top-down attention mechanism may strengthen the robustness of the vision system (Luo et al., 2015) and reduce energy consumption by suppressing firing rates of neurons (Martinez-Trujillo and Treue, 2004). Therefore, incorporating top-down attention into SNNs can further improve the performance and bio-plausibility of existing SNN models. Some work developed SNN models with top-down attention but the generation of the attention map is based on manual design (Wu et al., 2013) or restricted to simple models (Arena et al., 2012), leading to less flexibility and scalability. Direct learning of top-down attention maps is desired for building practical SNN models with top-down attention. However, the difficulties of acquiring sufficient labeled attention maps and designing effective supervision objectives hinder its development.

In this work, we report a hybrid network model that enhances a feedforward SNN with top-down attention (HTDA). The top-down attention maps are generated based on the features extracted by the SNN. Although an SNN with multiple spikes to approximately represent real numbers and trained by surrogate gradient descent can be used to complete regression tasks such as attention generation, the overall performance still lags behind the ANN counterpart (Zhao et al., 2022). To alleviate the difficulty of optimization and improve the performance, an ANN is deployed to generate the attention maps because it has more accurate representation and can be easily optimized by mature gradient-based methods. The generated attention map then modulates the encoding layer of the SNN. Thus, the hybrid model forms a closed loop to attend to the most informative inputs, making it possible to improve the robustness and energy efficiency of the SNN through iterative inferences. Furthermore, to address the direct learning of the top-down attention, we propose a general principle and a corresponding weakly-supervised objective for the attention map. With this objective, manual annotations are not required for training the attention map generator. Thus, the hybrid model can be optimized in an end-to-end manner with surrogate gradient descent. We use object recognition tasks to investigate the HTDA model, and demonstrate stronger robustness to adversarial noise, higher computing efficiency, and better interpretability. On the CIFAR-10, CIFAR-100, and MNIST benchmarks, the HTDA model reduces firing rates by up to 50% and improves adversarial robustness by up to 10% with better accuracy compared with the state-of-the-art SNNs. The HTDA model is also verified on the dynamic N-MNIST dataset and achieves consistent improvements. This work comprehensively studies the potential benefits of ANNs-based top-down spatial attention to SNNs on challenging machine learning tasks. With a new objective to optimize the proposed attention mechanism, the HTDA model provides a strong baseline and sheds light on SNNs enhanced by top-down attention using a hybrid approach. Our contributions are summarized as follows:

1. We develop a hybrid network model with top-down attention, which is composed of an SNN to extract spatiotemporal features and an ANN to generate top-down attention maps. The hybrid model combines the strengths ANNs and SNNs.
2. We propose a general principle and a corresponding weakly-supervised objective for generating attention maps, which facilitate direct learning of the top-down attention without manual annotations.
3. We investigate the HTDA model on object recognition tasks and demonstrate that it has greater robustness to adversarial noise, higher computing efficiency, and better interpretability compared with the state-of-the-art SNNs.

## 2. Materials and methods

In this section, we introduce the design philosophy and the overall architecture of the hybrid model with top-down attention. The formulation of the adopted neuronal models and the training method of the hybrid model are presented. More importantly, we provide the design principle and the corresponding objective for the top-down attention.

### 2.1. Overall architecture

**Figure 1:**
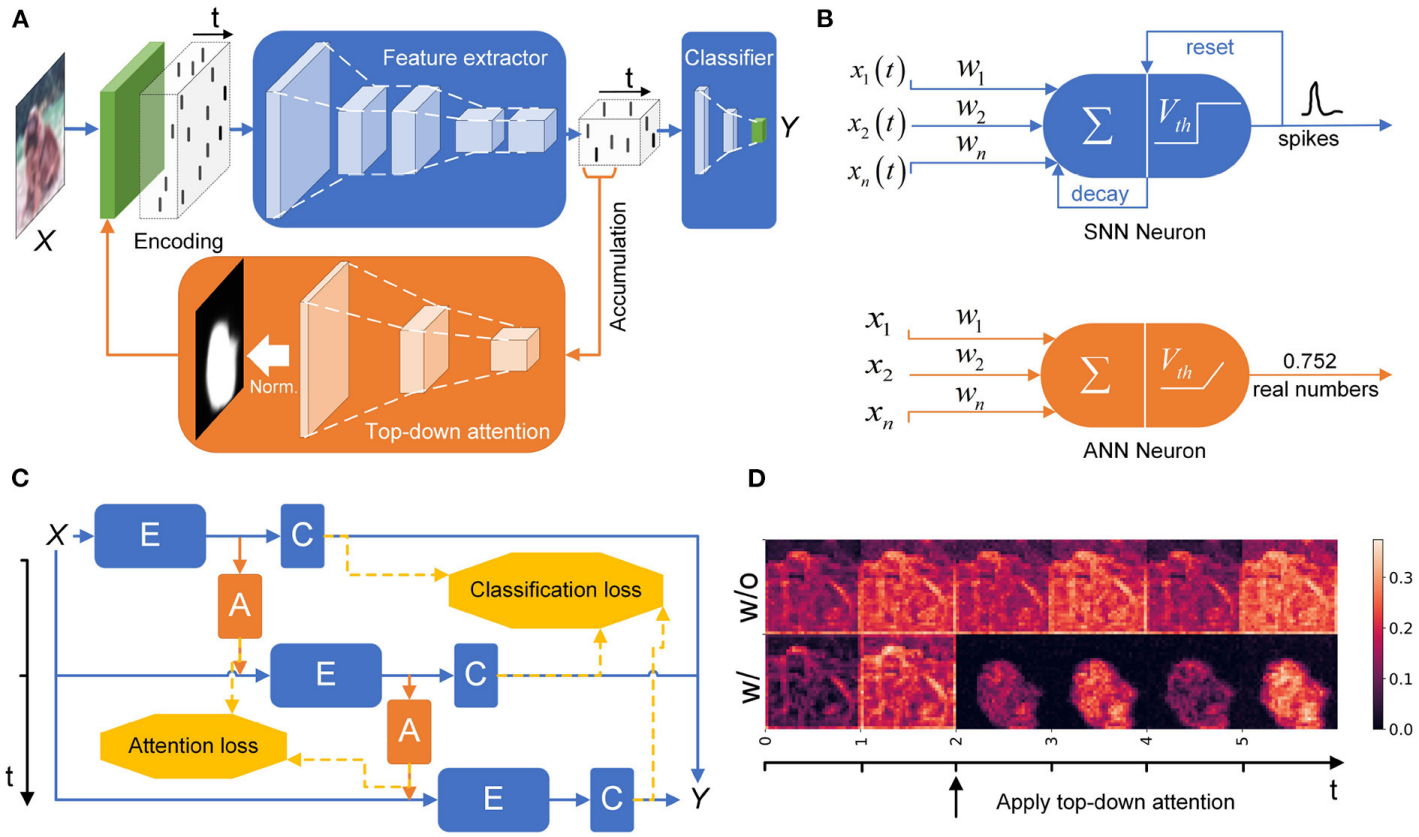
**(A)** The schematic of the HTDA model. The SNN-based feature extractor generates a spatiotemporal representation, which is then fed into an ANN to generate top-down attention maps and a classifier to give predictions. The attention map modulates the encoding layer of the SNN. **(B)** Neuronal models used in the HTDA model. **(C)** Training methods of the HTDA model. The HTDA model is jointly trained by unrolling in the time dimension. A weakly-supervised objective is designed to optimize the attention map generator. **(D)** Firing rates of the encoding layer of the feature extractor with or without top-down attention. The firing rate is calculated as the proportion of firing neurons in different channels at a certain spatial location at a certain time step.

The overall architecture of the HTDA model is inspired by the top-down attention structure of the perceptual system in the human brain (Baluch and Itti, 2011). The backbone of the HTDA model is a convolutional SNN, which is used to extract spatiotemporal features and perform downstream tasks. Besides, to alleviate the difficulty of optimization, an ANN is deployed to generate top-down attention maps based on the extracted features because ANNs have accurate real-valued representation. This configuration can also improve training stability and efficiency. The generated attention map then modulates the behavior of the feedforward SNN, thus forming a closed loop to give more reliable inference. The overall architecture of the hybrid model is illustrated in Figure 1A. Stimulated by the input ***X***(*t*) and modulated by the attention map ***M***_*n*_, the SNN-based feature extractor *E*(·) generates a time-varying signal ***F***(*t*), which represents the input in the spatiotemporal domain. The processing of the feature extractor can be formulated as the following equation:


(1) $$F(t)=E(X(t)\times M_{n},\theta_{E},h_{E}),\;t\in (nT_{f},(n+1)T_{f}],n\in N$$

where **θ**_*E*_ denotes learnable parameters of *E*(·) and ***h***_*E*_ denotes the initial value of the state variables of *E*(·). *T*_f_ denotes the period of generating attention maps, which can be determined by the frequency of input signals. For instance, the inputs that change rapidly should have a small *T*_f_. The first layer of the SNN is an encoding layer, which learns to encode inputs to spike trains.

On the one hand, the signal ***F***(*t*) will be then fed into the SNN-based classifier *C*(·) to give predictions, such as categories in recognition tasks. To make the prediction more reliable, the outputs of the classifier at each sampling time are concatenated and then processed by a learnable decoder to give the final result. As a generalization of averaging the prediction over the time dimension, the learnable decoding scheme of the concatenated features can lead to better performance due to adaptive feature selection and high representation precision (Wu et al., 2019). This process is formulated as follows:


(2) $$\{\;\begin{array}{l} F_{C}(t)=C\left(F(t),\theta_{C},h_{C}\right)\\ Y=w_{C}^{T}{\left[F_{C}{\left(T_{\text{d}}\right)}^{T},F_{C}{\left(2T_{\text{d}}\right)}^{T},...,F_{C}{\left(KT_{\text{d}}\right)}^{T}\right]}^{T}+b_{C} \end{array}$$

where ***Y*** is the final prediction of the recognition. Similarly, **θ**_*C*_ denotes learnable parameters of *C*(·) and ***h***_*C*_ denotes the initial value of the state variables of *C*(·). ***w***_*C*_ and ***b***_*C*_ are parameters of the linear decoder. *T*_d_ is the sampling interval and *K* is the number of sampling in an inference. For practical applications, *T*_d_ can be set as 1ms according to widely adopted settings (Wu et al., 2022). Therefore, *K* is determined by the duration of input signals.

On the other hand, the signal ***F***(*t*) will be integrated over a fixed period *T*_f_ and then passed into the ANN to generate the top-down attention map ***M***_*n*_. ***M***_*n*_ is a matrix whose size is equal to the spatial size of the input. The attention map will modulate the encoding layer of the SNN-based feature extractor, promoting the SNN to concentrate on the most informative part of the input. The modulation process can be implemented by multiplying the input with the attention map. The generation of the attention map can be formulated as the following equation:


(3) $$\begin{array}{l} M_{n}=\text{Sigmoid}\\ \;\left\{T_{\text{e}}\times \text{LayerNorm}\left[A\left(\frac{1}{T_{\text{f}}}\int_{(n-1)T_{\text{f}}}^{nT_{\text{f}}}F\text{d}t,\theta_{A}\right)\right]\right\} \end{array}$$

where **θ**_*A*_ denotes learnable parameters of *A*(·). The initial value of the attention map, ie, ***M***_0_, is set to an all-ones matrix ***J***, indicating no preference for each part of the input. Layer normalization (Ba et al., 2016) and the sigmoid function with a temperature coefficient *T*_e_ are applied for the attention map. The temperature coefficient determines the slope of the sigmoid curve, and thus determines the range and magnitude of the non-zero gradient of the sigmoid function. In the inference phase, large *T*_e_ is desired because it can improve the hardness or concentration degree of the attention map. However, large *T*_e_ can result in gradient vanishing or explosion (Han and Moraga, 1995). To enlarge the range of the non-zero gradient and thus alleviate gradient vanishing, the temperature coefficient increases gradually from 1 to a large value in the training phase and then fixed in the inference phase. This scheduling strategy of the temperature coefficient is critical for stable training of the HTDA model.

Notably, the configuration of the ANN-based attention map generator has unique advantages. A lot of neuroscience studies have revealed that the top-down feedback signals in perceptual pathways have different timescales from bottom-up sensory inputs (Larkum et al., 2004; Sarter et al., 2016; Helfrich et al., 2019). The top-down signals that reflect internal goals or working memory vary relatively slowly (Egeth and Yantis, 1997). With a relatively large time scale, rate-coding SNNs can be adapted to generate attention maps for the proposed model, which can use multiple spikes over a time window to approximately represent real numbers. However, the performance of such SNN on regression tasks is still inferior to the ANN counterpart (Zhao et al., 2022). Besides, multiple iterations of the SNN result in lower computational efficiency. To address this issue, we use an ANN to generate attention maps. On the one hand, the ANNs can be considered as the time-averaged version of rate-coding SNNs and naturally have real-valued representation (Deng et al., 2020), whose optimization methods have been investigated more deeply (LeCun et al., 2015). On the other hand, the ANN-based attention map generator reduces computation cost by only iterating once in the period *T*_f_. With the proper *T*_f_, the change of the input during the period is small, and thus the attention map generated based on the integrated features can track the focused object in the input. The quality of the attention map can also be improved due to the high precision of the feature integration and the ANN representation. Notably, this discussion does not lead to a conclusion that SNNs are fundamentally inferior to ANNs in performing attention. On the contrary, the unique characteristics of SNNs can be further investigated to implement various attention mechanisms (Chen and Gong, 2022). In this work, popular ReLU-based ANN neurons are adopted in the implementation of the attention generator.

### 2.2. Neuronal models

As the backbone of the HTDA model, the feedforward SNN serves as the key component for processing sensory inputs. SNNs have the characteristics of intrinsic temporal dynamics, sparse computation, and event-driven updates, which are appropriate for processing spatiotemporal information. In the HTDA model, we adopt the prevalent leaky integrate-and-fire (LIF) model for SNNs. The LIF model is easy to be implemented with moderate bio-plausibility. It should be noted that the proposed HTDA model is neuronal model-agnostic and not restricted to the specific LIF model. The schematic of the neuronal models used in this work is presented in Figure 1B. The equation of the membrane potential of the LIF model is presented as follows:


(4) $$\begin{array}{r} \tau \frac{\text{d}u\left(t\right)}{\text{d}t}=-\left(u\left(t\right)-u_{\text{reset}}\right)+RI\left(t\right) \end{array}$$

where *u*(*t*) is the membrane potential and *u*_reset_ is the reset potential. τ is the time constant and *R* is the resistance of the membrane, respectively. *I*(*t*) is the afferent current of the neuron, including pre-synaptic inputs and bias currents. If the membrane potential *u*(*t*) exceeds a threshold *V*_th_, the neuron will emit a spike and reset its membrane potential. For better simulation, the equation can be discretized in time by the numerical method (Wu et al., 2018). The iterative formula of the membrane potential of a layer of neurons with a sampling interval *T*_d_ is presented as follows:


(5) $$\{\begin{array}{ll} & h_{k+1}=h_{k}\lambda \left(1-o_{k}\right)+w*x_{k+1}\\ & o_{k}=H\left(h_{k}-V_{\text{th}}\right)\\ & \lambda ={\text{e}}^{-\frac{T_{\text{d}}}{\tau }} \end{array}$$

where *k* is the index of the time step and ***o***_*k*_ is the spiking output. λ is the decay factor of the membrane potential, usually ranging from 0 to 1. *H*(*x*) is the Heaviside step function, whose surrogate gradient can be approximated by a window function. ***w*** is the weight of the synapses and ***x***_*k*_ is the pre-synaptic spiking input. * denotes a certain linear operation, such as vector multiplication and convolution. The decay factor λ and the threshold *V*_th_ of the HTDA model are learnable to better support the generation of the attention map. In one inference, as mentioned above, the SNN model iterates *K* steps to give the final prediction.

### 2.3. Training method

The ANN and the SNN in the HTDA model are jointly trained by unrolling in the time dimension, which is illustrated in Figure 1C. The unrolled network forms a directed acyclic graph, which can be optimized based on gradient descent. The non-differential property of the spike generation can be solved by surrogate gradient (Neftci et al., 2019). The overall minimization objective is formulated as the following equation:


(6) $$ℒ=\alpha {ℒ}_{C}+(1-\alpha ){ℒ}_{M}$$

where $L_{C}$ is the loss for object recognition and $L_{M}$ is the loss for generating the attention map. α is a hyper parameter that adjusts the weight of the two terms. $L_{C}$ can be implemented by the softmax cross-entropy loss.

Generally, if we have sufficient ground-truth labels of the attention maps, $L_{M}$ can be easily implemented as the mean-square-error objective. However, these labels are difficult to obtain and the manual annotations are not necessarily optimal. To address this issue, we propose a general principle for generating these attention maps. On this basis, we design a feasible weakly-supervised objective without the need of detailed annotations. This approach facilitates the direct learning of the top-down attention, which not only reduces manual intervention but also improves flexibility.

Inspired by neuroscience studies, the proposed principle of designing $L_{M}$ is to promote the HTDA model to utilize an integral and small subset of the input to give accurate predictions. In other words, the attention map should be sparse, diverse, and smooth. At meanwhile, the attention map should not degrade the classification accuracy of the HTDA model. Guided by this principle, a feasible formulation of $L_{M}$ is presented as follows:


(7) $$\begin{array}{l} {ℒ}_{M}=\frac{1}{NP}\sum_{n=1}^{N}\{\gamma \|M_{n}{\|}_{1}-\beta \|M_{n}-mean(M_{n}){\|}_{2}^{2}\\ \;+(1-\gamma -\beta )\|M_{n}*K{\|}_{1}\} \end{array}$$

where *P* is the number of elements of the attention map. *N* is the number of generated attention map in an inference, which can be calculated as $\left\lfloor \frac{K*T_{\text{d}}}{T_{\text{f}}}\right\rfloor$. K is a kernel of a high pass filter, such as the Laplacian operator. mean(·) is an operator to calculate the mean value of all elements of a given matrix.

The first term of $L_{M}$ promotes the ANN to generate sparse attention maps. Due to the non-differentiability of *L*_0_ norm, *L*_1_ norm is adopted as the optimal convex approximation of the *L*_0_ norm for promoting sparsity. At the same time, constrained by the classification loss, the generated map should not be too sparse to degrade the accuracy. The second term is used to maximize the variance of all elements of the attention map. This objective can promote the values of all elements toward 0 or 1, thus improving diversity. Otherwise, all the elements of the attention map may tend to be 0.5, leading to loss of selectivity. The third term promotes the attention map to be smooth, which can make the focused object integral and improve training stability. γ and β are coefficients that balance these three terms. With this weakly-supervised objective, the HTDA model can be optimized in an end-to-end manner, resulting in greater flexibility.

As a comparison of the top-down attention investigated in this work, stimulus-driven bottom-up attention relies on the input to produce saliency maps, which is mainly implemented in the feedforward pathway and usually has a relatively small time scale (Egeth and Yantis, 1997; Itti and Koch, 2001). On the contrary, the attention mechanism in the HTDA model is not only based on the input stimulus but also guided by the goal of accurate object classifications in the training phase. In the inference phase, the classification goal implicitly guides the attention through iterative inference. Additionally, the attention maps are generated based on high-level information represented in deep layers of the SNN. Feedback connections are introduced to apply attention to the spike encoder. In this manner, the HTDA model forms a closed loop to give accurate and robust predictions over a time window, whose attention has a relatively large time scale. Therefore, the attention in the HTDA model is more like a type of top-down attention.

## 3. Results

In this section, we conduct experiments of the HTDA model on object classification tasks. The experimental results demonstrate that the HTDA model can generate desired top-down attention maps according to the design principle. The attention maps are robust to random translation, rotation, and noise. With the assistance of the attention map, the HTDA model achieves strong robustness to adversarial noise and reduces computation cost. Additionally, the generated attention map also improves interpretability, which can help to debug the model by presenting the focus of the feature extractor. The HTDA model is also evaluated on neuromorphic datasets and achieves consistent improvements. The experimental settings are summarized as follows. Please refer to the code for more details https://gitee.com/circle-pass-filter/htda.

**Network**. The SNN-based feature extractor is a 6-layer VGG (Simonyan and Zisserman, 2014)-like convolutional network and the ANN-based attention map generator is a 3-layer deconvolutional network (Figure 1A). In the following sections, we use VGG-*x*c to denote the VGG-like network with *x* channels in the first layer. The classifier is a fully connected network with one hidden layer. The baseline model used for comparison (S-SNN) is composed of the same SNN-based feature extractor and classifier without the top-down attention structure, whose thresholds and decay factors are fixed according to the state-of-the-art training settings (Wu et al., 2022). In the experiments, *T*_f_ is set to 2*T*_d_. The number of iterations *K* is set to 6 for static data and 10 for neuromorphic data. To improve efficiency, the attention map is generated once for static inputs and shared by subsequent iterations. Synaptic weights of neurons are initialized according to Kaiming's uniform initialization scheme (He et al., 2015). Membrane potentials of the LIF neurons are initialized as zero. Following widely-adopted settings in the machine learning community, one LIF neuron can have both excitatory synapses and inhibitory synapses at the same time (Wu et al., 2018).

**Dataset**. The effectiveness of the HTDA model is verified on MNIST (LeCun et al., 1998), CIFAR-10 (Krizhevsky et al., 2009), CIFAR-100, and N-MNIST (Orchard et al., 2015) datasets. CIFAR-10 contains 10 classes of 32 × 32 colorful images, with 50k samples for training and 10k samples for test. CIFAR-100 is similar to CIFAR-10 but contains 100 classes. MNIST has 60k training samples and 10k test samples, whose spatial size is adjusted to 32 × 32. N-MNIST is the neuromorphic version of MNIST, which is generated based on dynamic vision sensors. Random flipping and cropping are used for data augmentation in the training phase.

**Training**. The HTDA model is jointly optimized by mini-batch stochastic gradient descent with a momentum of 0.9. The batch size is set to 200 and the initial learning rate is set to 0.1. To improve training stability, warm-up approach (He et al., 2016) is adopted. By grid search of the hyper parameters, β and γ are set to 0.40 and 0.51 for all datasets, respectively. α is set to 0.10 for CIFAR-10 and CIFAR-100, and 0.01 for MNIST and N-MNIST. The maximum of the temperature coefficient of the attention map generator is set to 6 for all experiments.

### 3.1. Improved interpretability

**Figure 2:**
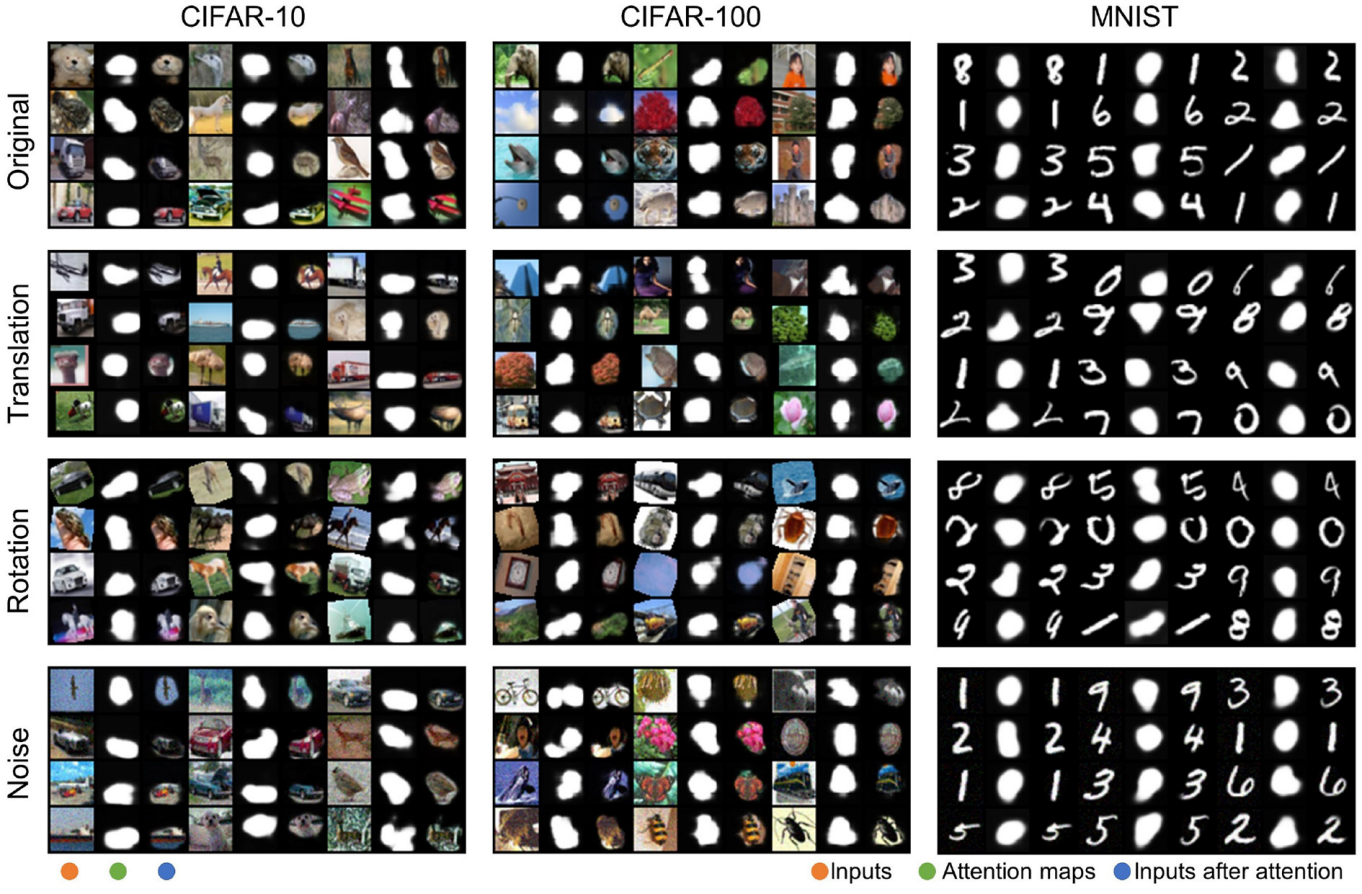
Attention maps for samples in CIFAR-10, CIFAR-100, and MNIST under different settings, respectively. The images are arranged in columns for the input, the corresponding attention map, and the input after attention.

Attention maps indicate the focus of the network model, which can improve interpretability. As shown in Figure 2, we present the attention maps generated by the HTDA model for samples in CIFAR-10, CIFAR-100, and MNIST. The feature extractor is a VGG-128c network. The experimental results demonstrate that the ANN can generate proper attention maps, which are smooth, diverse, and sparse. The object outlined by the attention map is integral and well-aligned with our expectations about the salient features. Powered by the attention map, the HTDA model promotes the SNN to focus on the most informative subset of the input and filter out background information. Additionally, we present the attention map for the input with random translation and rotation, and the input perturbed by Gaussian noise, respectively. The ANN can also generate desired attention maps for these transformed inputs. It is worth noting that the training samples are not augmented by random rotation and adding noise, which demonstrates the robustness and adaptivity of the HTDA model.

**Figure 3:**
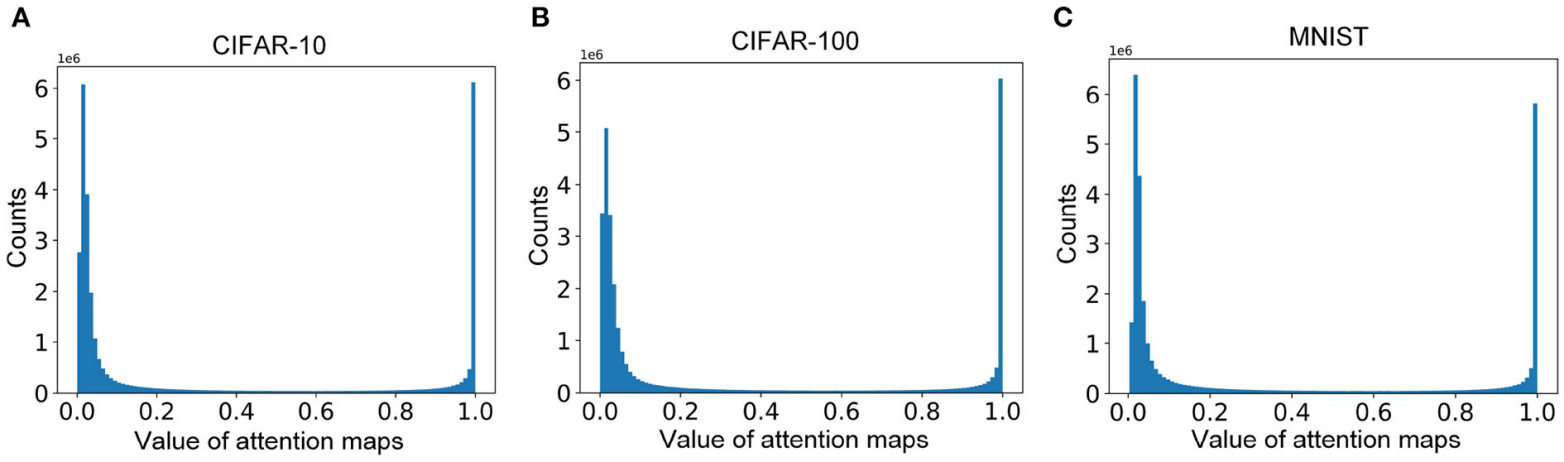
**(A–C)** The distributions of the attention maps for samples in CIFAR-10, CIFAR-100, and MNIST, respectively.

The distributions of the attention maps for samples in CIFAR-10, CIFAR-100, and MNIST are presented in Figure 3. The values of most elements of the attention map are near 0 and 1. These results indicate the attention maps are sparse and the top-down attention works in an all-or-none manner, which is consistent with the weakly-supervised objective (Equation 7). The distributions are beneficial to improving the diversity of the attention map, leading to better selectivity.

### 3.2. Improved robustness

In this section, we evaluate the robustness of the HTDA model with different capacities on CIFAR-10, CIFAR-100, and MNIST datasets, and compare with that of the baseline SNN model. The adversarial robustness is evaluated by the project gradient descent (PGD) attack method (Madry et al., 2018), which uses multi-step gradient ascent to calculate adversarial perturbation. The adversarial perturbation in these experiments is bounded by *L*_∞_ norm. The PGD method is a popular adversarial attack method for evaluating model robustness. The input perturbed by the PGD method is calculated as the following iterative formula:


(8) $$\begin{array}{c} X_{0}=X+\text{Uniform}\left(-\epsilon ,\epsilon \right)\\ X_{i+1}=P_{S}\left[X_{i}+\eta \cdot \text{sgn}\left(\nabla_{X}ℒ\left(Y,\hat{Y}\right)\right)\right] \end{array}$$

where ***X***_*i*_ denotes the perturbed input and ϵ denotes the perturbation budget. sgn(·) is the sign function. The perturbation budget is the maximum *L*_∞_ norm of the adversarial perturbation, which indicates the intensity of the adversarial attack. Generally, larger perturbation leads to lower accuracy. The step size η is set to 2/255 for CIFAR-10 and CIFAR-100, and 20/255 for MNIST, respectively. The number of iterations is set to 10. Ŷ is the ground truth of the prediction. *P*_*S*_(·) is an operator to project the perturbed input into the image space. Uniform(−ϵ, ϵ) generates a uniform noise matrix to initialize the adversarial perturbation, whose size is equal to the input. Notably, the adversarial perturbation is generated for the whole model. Thus, the attention mechanism is also considered when calculating gradients for generating adversaries. The PGD method only requires the sign of the gradient to generate adversarial perturbation, which is relatively insensitive to the exact value of the surrogate gradient in SNNs. Therefore, gradient-based methods for evaluating adversarial robustness have been adopted by the SNN community (Sharmin et al., 2020; Kundu et al., 2021; Kim et al., 2022; Nomura et al., 2022).

The reported robustness is measured by the accuracy of the model for adversarially perturbed inputs. In the experiments, three different perturbation budgets, ϵ_1_, ϵ_2_, and ϵ_3_, are adopted to evaluate the model robustness. For CIFAR-10 and CIFAR-100, ϵ_1_, ϵ_2_, and ϵ_3_ are set to 1/255, 2/255, and 3/255, respectively. For MNIST, ϵ_1_, ϵ_2_, and ϵ_3_ are set to 20/255, 40/255, and 60/255, respectively. The experimental results and corresponding standard deviations under different settings are summarized in Table 1. Every experiment is run three times under the same setting except for random seeds.

**Table 1:** Comparison of accuracy and robustness under different settings (%).

**Settings**		**CIFAR-10**		**CIFAR-100**		**MNIST**	
		**S-SNN**	**HTDA**	**S-SNN**	**HTDA**	**S-SNN**	**HTDA**
VGG-32c	Clean	87.88 ± 0.12	**88.57 ± 0.03**	59.73 ± 0.14	**62.07 ± 0.05**	99.14 ± 0.06	**99.25 ± 0.07**
	ϵ_1_	66.23 ± 0.28	**72.20 ± 0.34**	37.02 ± 0.39	**46.00 ± 0.27**	95.84 ± 0.14	**96.03 ± 0.06**
	ϵ_2_	31.81 ± 0.70	**43.56 ± 0.81**	15.26 ± 0.51	**26.15 ± 0.15**	72.65 ± 3.72	**79.52 ± 0.66**
	ϵ_3_	10.23 ± 0.89	**19.77 ± 0.56**	5.64 ± 0.16	**12.46 ± 0.29**	9.03 ± 2.51	**23.07 ± 0.24**
VGG-64c	Clean	90.37 ± 0.09	**90.93 ± 0.10**	64.61 ± 0.43	**66.09 ± 0.07**	99.17 ± 0.03	**99.22 ± 0.10**
	ϵ_1_	72.58 ± 0.28	**75.44 ± 0.60**	39.93 ± 0.27	**45.80 ± 0.39**	96.1 ± 0.11	**95.95 ± 0.11**
	ϵ_2_	42.01 ± 0.39	**47.11 ± 1.20**	16.31 ± 0.33	**22.86 ± 0.76**	72.08 ± 1.24	**79.00 ± 0.58**
	ϵ_3_	17.32 ± 0.16	**21.84 ± 0.95**	5.91 ± 0.25	**10.20 ± 0.44**	9.98 ± 0.37	**25.57 ± 2.41**
VGG-128c	Clean	90.83 ± 0.21	**91.99 ± 0.17**	68.17 ± 0.31	**69.13 ± 0.06**	99.14 ± 0.04	**99.19 ± 0.04**
	ϵ_1_	75.36 ± 0.54	**79.25 ± 0.24**	44.69 ± 0.42	**48.25 ± 0.20**	96.02 ± 0.17	**96.03 ± 0.11**
	ϵ_2_	49.13 ± 0.60	**56.51 ± 0.36**	21.69 ± 1.08	**25.55 ± 0.72**	74.44 ± 2.32	**78.96 ± 0.55**
	ϵ_3_	25.38 ± 1.10	**31.25 ± 0.90**	8.90 ± 0.53	**11.62 ± 0.78**	14.82 ± 1.85	**28.42 ± 3.72**

The results of the HTDA model are marked in bold.

The experimental results show that the robustness of the HTDA model is improved significantly than the baseline SNN in most cases. On CIFAR-10 and CIFAR-100, the mean robustness of the HTDA model exceeds that of the baseline by 3% to 10% under the perturbation budgets of ϵ_1_, ϵ_2_, and ϵ_3_. On MNIST, the robustness of the HTDA model is improved by up to 15% under large perturbation budgets such as ϵ_3_. It should be noted that the standard deviation of the reported robustness is about 0.5% and substantially smaller than the difference of mean values. These results indicate that the improvement achieved by the HTDA model is statistically significant. Interestingly, with a relatively small network such as the VGG-32c, the robustness improvement of the HTDA model on CIFAR-10 and CIFAR-100 is larger than that with a large network. These results are consistent with the hypothesis that the HTDA model can allocate the limited capacity to process the most informative subset of the input. Thus, the HTDA model can achieve stronger robustness with a small network capacity.

The HTDA model improves robustness because that it can filter out background information by suppressing the activation of corresponding encoding neurons of the SNN. This approach reduces the dimension of the input space, therefore reducing the space of the potential adversarial examples to fool the network (Simon-Gabriel et al., 2019). Several works have demonstrated that elimination of the background of inputs using hand-designed attention masks prior to classification can improve robustness (Vaishnavi et al., 2020). In contrast, the HTDA model can automatically generate attention maps based on the extracted features without manual annotations.

The above explanation can be verified by the comparison in Table 1. Interestingly, the experiments on MNIST serve as an ablation study. Samples of the digits in MNIST have black backgrounds, which can be seen as the results after attention to a certain extent. Therefore, the robustness improvement of the HTDA model on MNIST should not be significant. This inference is consistent with the presented results. Specifically, with the network of VGG-32c and under the perturbation budget of 20/255, the robustness of the HTDA model is 96.08%, which is comparable to that of the baseline (95.69%). On the other hand, under larger perturbation budgets, the robustness improvement increases because the HTDA model has better efficiency of the network capacity.

### 3.3. Improved efficiency

**Figure 4:**
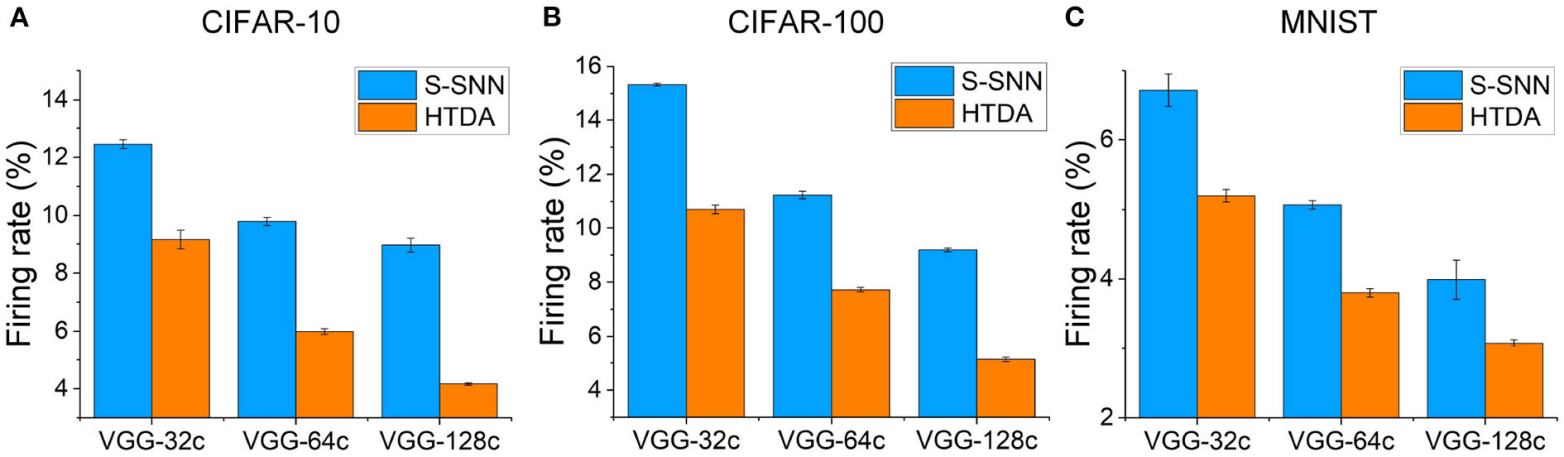
**(A–C)** Mean firing rates of the feature extractors on CIFAR-10, CIFAR-100, and MNIST, respectively. The mean firing rate is calculated as the proportion of firing neurons in all layers over all time steps.

The HTDA model can reduce firing rates of the SNN-based feature extractor, resulting in lower communication and computation costs, and thus improved energy efficiency. In the human brain cortex, it is estimated that the energy consumed by communication is about 35 times larger than that of computation (Levy and Calvert, 2021). The communication of spikes also poses challenges in terms of network bandwidth, energy consumption, and synchronization for neuromorphic systems (Painkras et al., 2013; Benjamin et al., 2014; Schuman et al., 2017). Thus, reducing firing rates of SNNs can improve energy efficiency significantly. Here, we evaluate the firing rates of the SNN-based feature extractor of the HTDA model after applying top-down attention and compare with that of the baseline SNN. The results are presented in Figure 4. The error bar shows the standard deviation of the firing rates of three independent trials. Across different datasets and network capacities, the firing rates of the SNN in the HTDA model decrease by a large margin than that of the baseline. The larger the network capacity, the more significant the decrease in firing rates. Specifically, the firing rate of the HTDA model with the VGG-128c network on CIFAR-10 decreases by 50%. This is because the HTDA model allocates as little as possible but sufficient resources to process information even with larger network capacities.

**Figure 5:**
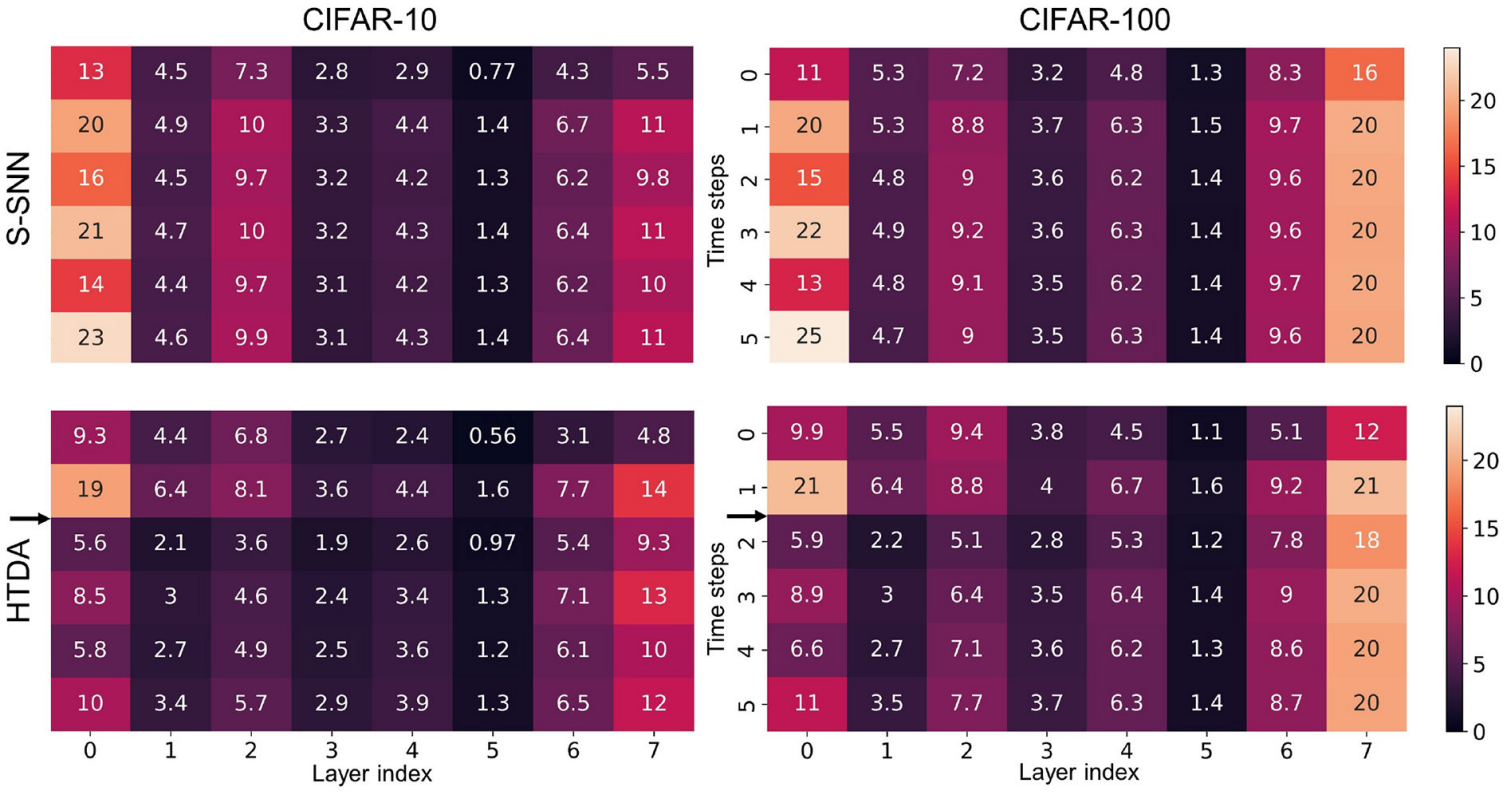
Firing rates of each layer of the feature extractor (VGG-128c) at different time steps under different settings. The firing rate presented in each cell of the matrix is the proportion of the firing neurons in a certain layer at a certain time step. On both CIFAR-10 and CIFAR-100, the firing rates of the HTDA model after attention decrease significantly.

As shown in Figure 1D, the firing rates of the neurons in the encoding layer of the HTDA model corresponding to the background of the input are significantly suppressed by the top-down attention. This does not degrade accuracy because the background contributes little to accurate and robust recognition of objects. The spatial firing patterns of subsequent layers are similar to the encoding layer, therefore reducing the firing rate of the entire HTDA model. These can be verified by the results of the firing rates of each layer of the feature extractor (Figure 5). The firing rates of the HTDA model decrease significantly after applying attention. As commonly accepted, neurons in deep layers represent the high-level abstraction of the input. Interestingly, the firing rates of the neurons in deep layers keep similar to the baseline. This phenomenon suggests that the HTDA model and the baseline SNN may have similar object-level representations of the input, and thus do not degrade accuracy on benign samples.

In the HTDA model, the ANN-based attention map generator brings additional computation cost and network parameters. For a fair comparison, we present the number of MACs (Multiply–Accumulate Operations) and parameters of the HTDA model on CIFAR-10 and compare with that of the baseline. The results are summarized in Table 2. The reported computing operations are estimated by considering firing rates and sparse computation. In other words, the neuron that does not fire will not lead to post-synaptic computation. Compared with the baseline, the computation costs of the HTDA model are reduced by up to 50%, which is similar to the results of the firing rates. Additionally, the parameters of the HTDA model are increased slightly than the baseline. Therefore, the additional computation cost brought by the ANN is much less than the reduced cost brought by suppressing firing rates. In total, the HTDA model can improve energy efficiency, especially by reducing the amount of spike emissions.

**Table 2:** Comparison of computing operations and the number of parameters of different models.

**Settings**		**VGG-32c**	**VGG-64c**	**VGG-128c**
MAC (M)	S-SNN	31.1	92.1	331.5
	HTDA	23.7	60.4	165.5
Para. (M)	S-SNN	2.94	5.32	13.52
	HTDA	3.10	6.55	16.14

### 3.4. Evaluation on neuromorphic data

**Figure 6:**
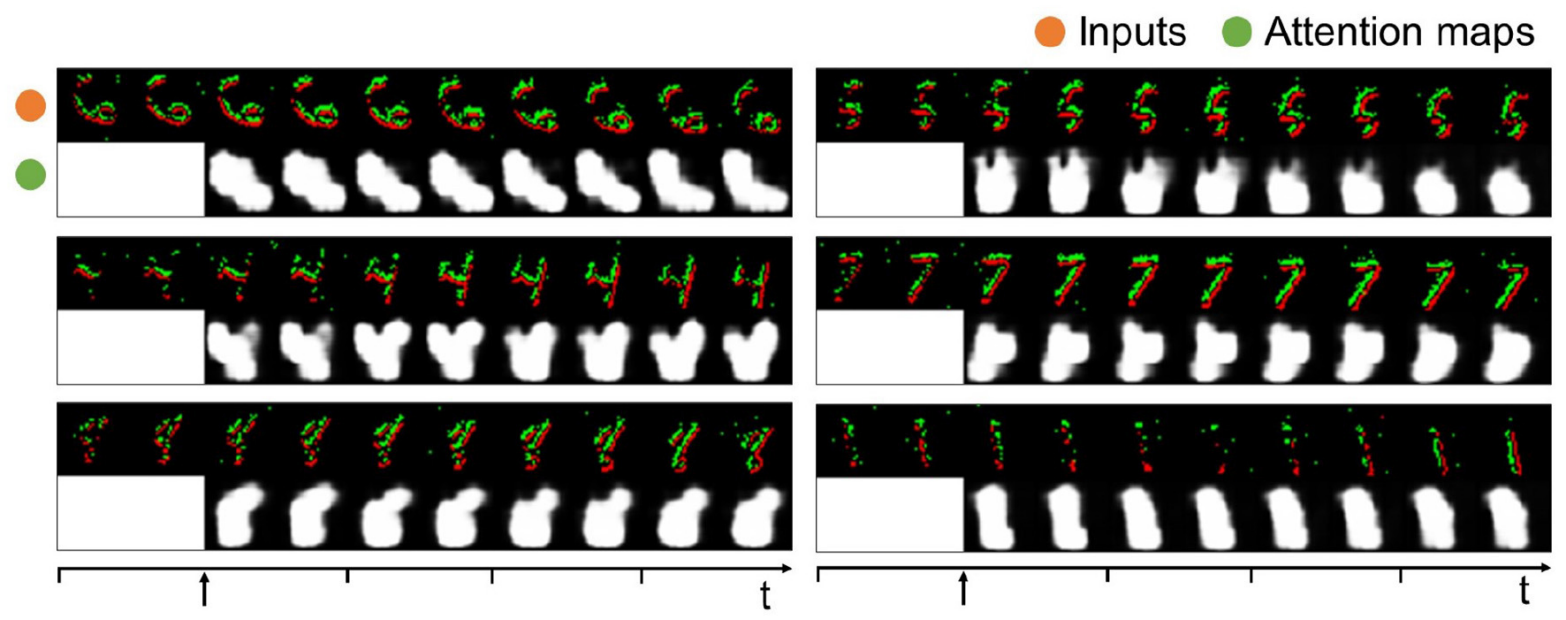
Event data of samples in N-MNIST and corresponding attention maps. The attention maps are set to all-ones matrices at the first two steps and updated every two steps.

We evaluate the HTDA model on a neuromorphic dataset, N-MNIST, to demonstrate its compatibility on dynamic data. To improve efficiency, the event data of N-MNIST is accumulated over a period of 5ms and 10 time steps are used to represent the samples. The digits in N-MNIST move in space. Therefore, the HTDA model is required to generate dynamic attention maps to track the digits, which is more challenging. As presented in Figure 6, the generated attention maps of the HTDA model on N-MNIST can dynamically concentrate on the moving digits. More importantly, as the time steps increase, the attention maps become more fine-grained. These results demonstrate the effectiveness of the HTDA model powered by the closed-looped iterative inference.

We further evaluate the robustness of the HTDA model on N-MNIST, whose evaluation protocol is similar to the experiments presented above. Notably, the adversarial noise is generated for the full model including the feedback attention mechanism. The results are summarized in Table 3. ϵ_1_, ϵ_2_, and ϵ_3_ are set to 10/255, 40/255, and 50/255, respectively. Every experiment is run three times independently. Instead of binary adversarial noise, the generated real-valued adversarial noise is directly added to the samples in N-MNIST, which is more reliable to evaluate the robustness. The results demonstrate that the HTDA model can improve robustness on neuromorphic data, which is consistent to the results of static datasets.

**Table 3:** Comparison of different models on N-MNIST.

**Settings**		**Robustness (%)**				**Para. (M)**	**MAC (M)**
		**Clean**	**ϵ_1_**	**ϵ_2_**	**ϵ_3_**		
VGG-32c	S-SNN	99.23 ± 0.11	98.99 ± 0.11	84.67 ± 3.63	44.89 ± 3.59	2.96	10.2
	HTDA	**99.29 ± 0.03**	**98.67 ± 0.11**	**92.52 ± 0.72**	**75.24 ± 6.22**	3.12	12.0
VGG-64c	S-SNN	99.19 ± 0.06	98.72 ± 0.02	46.68 ± 8.56	10.29 ± 3.62	5.91	34.5
	HTDA	**99.26 ± 0.05**	**98.61 ± 0.14**	**82.72 ± 2.86**	**55.08 ± 0.65**	6.57	40.4

The results of the HTDA model are marked in bold.

### 3.5. Ablation study

The top-down feedback attention mechanism and the learnable thresholds and decays are two key ingredients of the HTDA model. To investigate their effects on the overall performance of the HTDA model, we conduct an ablation experiment, in which the attention and the learnable are independently enabled or disabled. The experiment is conducted on CIFAR-10 with a VGG-32c network backbone. Training settings and the evaluation protocol are kept the same as that of the HTDA model. Results of the four settings are presented in Table 4. As indicated by the experimental results, these two key designs can both improve performance and reduce firing rates. The improvement independently achieved by the attention is more significant than that of the learnable thresholds and decays. Additionally, learnable thresholds and decays contribute more to the improvement of accuracy. In contrast, the attention mechanism contributes more to enhancing robustness and reducing firing rates. More importantly, these two designs can be combined to further improve the overall performance.

**Table 4:** Comparison of different settings on CIFAR-10 (%).

**Attention**	**Learnable**	**Accuracy**	**Robustness (ϵ_2_)**	**Firing rates**
*X*	*X*	87.88 ± 0.12	31.81 ± 0.70	12.45 ± 0.15
*X*	✓	88.09 ± 0.04	38.54 ± 0.40	11.20 ± 0.05
✓	*X*	87.84 ± 0.12	40.54 ± 0.68	8.48 ± 0.11
✓	✓	88.57 ± 0.03	43.56 ± 0.81	9.15 ± 0.32

To investigate the effect of the attention target on model performance, we conduct a contrast experiment on CIFAR-10 with a VGG-32c network, in which top-down attention maps are applied to the encoder and middle layers of the SNN. Except for the attention target, other settings of the model are the same as that of the HTDA model. Comparisons of accuracy, robustness under the perturbation budgets of ϵ_1_, ϵ_2_, and ϵ_3_, and firing rates are presented in Table 5. The experimental results show that the performance of these two models is comparable. Moreover, additional attention to middle layers can slightly improve robustness, especially under larger perturbation budgets. Generally, similar results of these two settings indicate that it is reasonable to only apply the attention to the encoder layer. Neurons in the encoder layer that are suppressed by the top-down attention will not trigger neurons in subsequent layers at the same spatial location. Therefore, it is not necessary to apply the top-down attention to the middle layers again. Nevertheless, more sophisticated attention mechanisms for middle layers are worth further exploring (van de Ven et al., 2020).

**Table 5:** Comparison of attention targets on CIFAR-10 (%).

**Targets**	**Accuracy**	**Robustness (ϵ_1_)**	**Robustness (ϵ_2_)**	**Robustness (ϵ_3_)**	**Firing rates**
Encoder	88.57 ± 0.03	72.20 ± 0.34	43.56 ± 0.81	19.77 ± 0.56	9.15 ± 0.32
Encoder&middle	88.52 ± 0.10	72.40 ± 0.68	45.05 ± 1.50	21.23 ± 1.13	9.33 ± 0.09

### 3.6. Comparison with state-of-the-art SNN models

In this section, we compare the HTDA model with the state-of-the-art SNN models on classification accuracy. Considering that the key innovation of the HTDA model lies in the architectural level instead of the network backbone level, we adopt the state-of-the-art SNN models with similar VGG-like backbones for comparison. The results are summarized in Table 6. The architecture of the VGG-128c in this work are similar to the CIFARNet and the VGG-16 network. The reported time steps are the maximum number of iterations in an inference and refer to the published work, which can be directly compared. The HTDA model exceeds the baseline S-SNN consistently and achieves state-of-the-art accuracy on CIFAR10 with fewer parameters and time steps. The accuracy of the HTDA model on CIFAR-100 is comparable to the state-of-the-art accuracy of converted SNNs with thousands of time steps. However, time steps of the HTDA model are much fewer than these converted SNN models because the HTDA model is directly optimized based gradient descent. In the future, more advanced backbones can be further combined in the HTDA model to achieve better performance.

**Table 6:** Comparison of accuracy on CIFAR-10, CIFAR-100, and MNIST.

**Dataset**	**Model**	**Backbone**	**Para. (M)**	**Steps**	**Accuracy (%)**
CIFAR-10	Unsupervised (Panda and Roy, 2016)	Spike CNN	-	250	75.42
	Back-propagation (Wu et al., 2019)	CIFARNet	45	8	90.53
	Converted SNN (Sengupta et al., 2019)	VGG-16	138	2,500	91.55
	Hybrid plasticity (Wu et al., 2022)	CIFARNet	45	12	91.08
	S-SNN (ours)	VGG-128c	13.52	6	**90.83 ± 0.21**
	HTDA (ours)	VGG-128c	16.14	6	**91.99 ± 0.17**
CIFAR-100	Converted SNN (Han et al., 2020)	ResNet-20	0.27	2,048	67.82
	Converted SNN (Han and Roy, 2020)	VGG-16	138	2,048	70.97
	S-SNN (ours)	VGG-128c	13.52	6	**68.17 ± 0.31**
	HTDA (ours)	VGG-128c	16.14	6	**69.13 ± 0.06**
MNIST	Converted SNN (Diehl et al., 2015)	Spike CNN	-	200	99.10
	Hybrid plasticity (Wu et al., 2022)	CIFARNet	45	12	99.50
	S-SNN (ours)	VGG-32c	2.94	6	**99.14 ± 0.06**
	HTDA (ours)	VGG-32c	3.10	6	**99.25 ± 0.07**

The results of our implementations are marked in bold.

## 4. Conclusion

In this work, we develop a hybrid neural network with a top-down attention mechanism. A general principle and a corresponding weakly-supervised objective are proposed for optimizing the top-down attention. The proposed HTDA model is investigated on object recognition tasks on CIFAR-10, CIFAR-100, MNIST and N-MNIST datasets. The experimental results demonstrate that the HTDA model can achieve strong robustness, high computing efficiency, and improved interpretability. Specifically, the HTDA model improves the robustness to adversarial noise by up to 10% and reduces the firing rates by up to 50% compared with the state-of-the-art SNNs. The accuracy of the HTDA model is also comparable to or larger than the state-of-the-art SNN models. This work promotes the development of the SNN models with top-down attention.

## Data availability statement

Publicly available datasets were analyzed in this study. This data can be found here: https://www.cs.toronto.edu/~kriz/cifar.html.

## Author contributions

FL designed the model and conducted experiments. RZ proposed the approach of enhancing spiking neural networks with the attention mechanism and supervised the project. Both authors contributed to the article and approved the submitted version.

## Funding

This work was partly supported by the National Nature Science Foundation of China (No. 61836004) and the National Key Research and Development Program of China (No. 2021ZD0200300).

## Conflict of interest

The authors declare that the research was conducted in the absence of any commercial or financial relationships that could be construed as a potential conflict of interest.

## Publisher's note

All claims expressed in this article are solely those of the authors and do not necessarily represent those of their affiliated organizations, or those of the publisher, the editors and the reviewers. Any product that may be evaluated in this article, or claim that may be made by its manufacturer, is not guaranteed or endorsed by the publisher.
